# Adolescents’ Feelings of Loneliness Considering Anxiety and Intrafamilial Relations

**DOI:** 10.3390/brainsci15121270

**Published:** 2025-11-26

**Authors:** Celina Timoszyk-Tomczak, Elżbieta Pieńkowska, Maria Ligocka, Marzena Piłat

**Affiliations:** Institute of Psychology, University of Szczecin, 71-017 Szczecin, Poland; elzbieta.pienkowska@usz.edu.pl (E.P.); maria.ligocka@usz.edu.pl (M.L.); marzena.pilat@usz.edu.pl (M.P.)

**Keywords:** loneliness, feeling of loneliness, intra-familial relations, adolescence, anxiety

## Abstract

**Background/Objectives**: Loneliness can be differentiated from social isolation. The first is the subjective perception of being isolated from others, while the other is the entire emotional and social experience. The feeling of loneliness defines the discrepancy between desired and actual social relations. Loneliness is an unpleasant phenomenon that involves quantitative and qualitative impoverishment of interpersonal relationships. The aim of this study was to analyze feelings of loneliness—including intimacy, social connections, and belonging—among adolescents and how these feelings relate to different aspects of family relationships, such as communication, cohesion, autonomy-control, and identity. **Methods:** The study was conducted among 136 adolescents (aged 15–17, 70% of girls and 30% of boys). The following self-report measures were used: the R-UCLA Loneliness Scale, the State–Trait Anxiety Inventory (STAI)—X2, and the Family Relations Questionnaire, version: My Family. Correlation analyses were performed, followed by stepwise regression for three loneliness sub-scales. **Results**: The results have shown that anxiety and adverse family relationship factors were identified as significant predictors of adolescent loneliness. Communication, cohesion, autonomy-control, and identity within the family context each contributed uniquely to the experience of loneliness. **Conclusions**: The conclusion is that trait anxiety and the evaluation of family functioning are very strong predictors of loneliness among young people. Moreover, distinct aspects of family relationships shape different forms of loneliness, highlighting the multifaceted nature of adolescent social experiences.

## 1. Introduction

Loneliness is an increasing mental health concern (depression, anxiety) in the adolescent population [1]. In Poland, in 2024, students and university attendees constituted the group with the highest intensity of loneliness—17% of respondents reported feeling lonely “very often” or “always” (CBOS, August 2024). Loneliness is multidimensional and interdisciplinary; it can be interpreted from different perspectives. It can be studied situationally and referred to as an individual condition, but it can also be treated as a relatively fixed set of psychological traits. Loneliness has positive and negative dimensions, which are reflected in its descriptions and typologies. Depending on the approach, it is also defined differently [2]. The cognitive-behavioral approach focuses on the measurable deficit and cognitive appraisal of loneliness, distinguishing its emotional and social dimensions [3]. Attachment theory emphasizes relational patterns underlying difficulties with closeness [4], while the phenomenological-existential perspective views loneliness as an inevitable aspect of existence, expressed as the experience of exclusion [2].

Loneliness can be differentiated from social isolation. The first is defined as the subjective perception of being isolated from others, while the other refers to the emotional and social experience as a whole [5]. Researchers additionally discern between social isolation and the feeling of loneliness and aloneness [6,7,8].

Loneliness differs from aloneness or solitude—aloneness means being with oneself, while loneliness involves a lack of contact with others and with oneself [9,10,11,12]. Typologies distinguish social loneliness (a sometimes positive need for solitude) and emotional loneliness (feeling abandoned or disappointed in relationships, often linked to anxiety and depression) [3,12,13]. The social aspect can be subjective or objective; emotional loneliness relates to isolation [14,15,16]. Studies among adolescents show that they define loneliness primarily through its emotional aspect, followed by its social and normative components [13,15,17].

In the present study, we focus on the concept of loneliness as defined by Russell, who describes loneliness as an unpleasant experience resulting from a perceived deficit in social relationships [3,18]. This theory offers a reliable operationalization [19] and emphasizes the cognitive aspect of loneliness, while also aligning with both social psychology and the psychology of individual differences.

The ecological model of human development clearly emphasizes that when analyzing loneliness, it is essential to consider both the intra-individual dimension and the multi-dimensional environmental influence on an individual’s development and functioning. From the perspective of social needs, different types of social relations satisfy different developmental needs over the course of a life [20,21].

From the developmental perspective, relationships with adults and peers are two main sources of both social development and the feeling of loneliness during adolescence [22]. Both social groups play different roles in an individual’s growth and fulfill different social needs. Various interpersonal relations are the source of potential developmental successes like attachment, belonging, and integration, as well as the source of values and support [23]. Peer-related loneliness may be evoked by unmet social needs that are fulfilled by adults and peers. It may result from the lack of physical opportunity to experience social relationships or the lack of social competencies that would allow a young person to have this type of experience. This situates the pre-source of loneliness felt by young people in the quality of relationships with their parents from the early stages of their development [24,25].

During adolescence, relationships with adults—especially within the family—are a potential source of important developmental effects (emotional, personality, and relational), and as these relationships evolve, parents play a key role in supporting autonomy and emotional independence [22,26,27,28,29,30]. Adolescence is a period of heightened risk for social isolation and perceived loneliness due to developmental changes and an increased need for physical distancing; however, solitude during this time may also support self-reflection, self-regulation, and creativity [11,29,31,32,33,34,35].

Many studies indicate that social relationships are an important source of psychological and physical well-being [6]. Research also confirms that loneliness is closely linked to well-being, and its strong correlates include depression and suicidal thoughts [7,36,37,38,39,40,41,42,43]. Importantly for the present study, social anxiety is considered a statistically significant variable related to the sense of loneliness during childhood and adolescence [41,42,44,45]. Lonely individuals typically display reduced social interest, higher shyness, lower self-esteem, and increased uncertainty [46]. Studies indicate that prosocial and aggressive behaviors, as well as factors such as gender, depression, shyness, and low self-esteem, are significant predictors of loneliness in youth [15,39,46,47,48,49,50]. Furthermore, data suggest that loneliness is not exclusively genetically determined [51], but is also associated with experiences and social patterns, including the quality and satisfaction of peer relationships [6,30,52,53,54,55,56,57].

Intrafamilial relations may become the direct source of perceived loneliness during different stages of personal development [58,59,60,61,62]. Research shows that key predictors of adolescent loneliness include low levels of social support and emotional expression from parents, lack of parental acceptance, and systemic family factors such as overprotection [63,64,65]. These findings highlight the importance of systemic aspects of family functioning—such as communication, cohesion, autonomy-control, and identity—in the context of youth mental health. Moreover, studies by Szcześniak and Tułecka [66] suggest that individuals who perceive their family of origin as cohesive, flexible, communicative, and satisfying tend to have a greater ability to process their own emotions (higher emotional intelligence), which leads to greater life satisfaction. Conversely, viewing the family as divided and chaotic may diminish the capacity for emotion management and result in lower life satisfaction.

Research findings indicate associations between the feeling of loneliness and internalizing disorders such as anxiety and depression [1,67,68]. At the same time, the quality of family relationships is an important predictor of the level of loneliness in adolescence [69,70]. While evidence supports the connections among these key factors, integrated models exploring their combined effects are still lacking.

The present study aimed to assess the intensity of loneliness among high school students and identify the main psychological and family factors influencing this phenomenon, with particular attention to anxiety and specific characteristics of intrafamilial relationships. We hypothesized that anxiety and family relationship components—specifically communication, cohesion, autonomy-control, and identity—would significantly contribute to explaining the variance in adolescent loneliness (H1). Furthermore, we expected that these aspects of family relationships would be significantly associated with loneliness (H2) and that anxiety would emerge as a key predictor of loneliness in adolescence (H3).

## 2. Materials and Methods

Standardized, psychometrically validated questionnaires were employed to assess key psychological constructs in adolescent participants. Data were collected in two secondary schools in Szczecin, which agreed to take part in the research project, with procedures following established ethical principles for psychological research.

### 2.1. Participants

The study was conducted on a sample of 136 individuals aged 15–17, including 104 girls (76%) and 32 boys (24%), all students of secondary schools in Szczecin. The sample consisted exclusively of first- and second-grade students, with final-year classes excluded to avoid disrupting exam preparations. Participation was open to all students who (along with their parents) provided written informed consent; no additional selection criteria were applied, and the observed gender imbalance resulted from voluntary consent. Due to this imbalance, no gender-specific analyses were performed.

Among participants, 100% reported having parents, 83.1% reported siblings, 97.1% reported grandparents, and 7.5% declared no close relationship with parents.

### 2.2. Procedure

Data collection took place in group sessions at schools, following instructions provided by the investigator and in their presence, allowing clarification of any questions from respondents. Participation was voluntary and anonymous, with written informed consent provided by all students and their legal guardians prior to inclusion. The study was conducted in a paper-and-pencil format and was not time-limited.

At the beginning of each session, participants completed demographic questions concerning age, gender, family structure, and close relationships. Following this, standardized self-report questionnaires were administered to assess psychological constructs of interest. Information concerning the study’s objectives, procedure, voluntary nature, and the right to withdraw at any stage was communicated. The research protocol received ethics approval from the University Ethics Committee.

Data collection took place in group sessions at schools, following instructions provided by the investigator and in their presence, allowing clarification of any questions from the respondents. All procedures emphasized the voluntary nature and anonymity of participation, with the possibility to withdraw at any stage of the study. The questionnaires included questions regarding basic family structure, with 100% reporting parents, 83.1% reporting siblings, 97.1% reporting grandparents, and 7.5% declaring no close relationship with parents. The study was not time-limited. The research protocol received ethics approval from the University Ethics Committee.

### 2.3. Instruments

Basic demographic data, including age, gender, and family structure, were collected prior to administering standardized psychometric questionnaires.

1. The R-UCLA Loneliness Scale (Revised University of California Los Angeles Loneliness Scale, [18]) adapted by Kwiatkowska, Rogoza, and Kwiatkowska [71] treats loneliness as a complex construct. The scale consists of 20 items that evaluate satisfaction and dissatisfaction with social relationships using a four-point response scale. The tool provides information about three spheres of individual functioning: Intimate Others (IOs), referring to the feeling of exclusion; Social Others (SOs), referring to the lack of closeness and support in relationships; and Belonging and Affiliation (BA), referring to the lack of community bonds.

The psychometric properties are valid: Cronbach’s alpha for the whole scale was 0.94, each item had a discriminating power higher than 0.40, and the model was reliable (CFI 0.95).

2. The State–Trait Anxiety Inventory (STAI) from the Polish adaptation by Spielberger et al. [72]. One of the two sub-scales was used X-2 (A) to measure anxiety-trait; that is, anxiety understood as a relatively stable personality trait. The questionnaire consists of 20 items, which the subject answers by selecting one of four pre-categorized answers. Both sub-scales have high internal consistency, with Cronbach’s alphas of 0.92. The stability of the X-2 scale is high, and while the X-1 scale is low due to the sensitivity of anxiety to change when measured as a state.

3. The Family Relations Questionnaire (Polish adaptation by Plopa and Połomski [73]) version: My Family was used to evaluate young persons’ perceptions of family relationships. The questionnaire consists of 32 items, each rated by the subject on a five-point scale. Family relationships are measured in the following dimensions: Communication (Com), Cohesion (Coh), Autonomy-Control (A-C), and Identity (I). Com measures the level of correct, open communication between the family members, which facilitates sharing problems and understanding from loved ones. Coh evaluates the strength of emotional bonds related to showing kindness, support, and acceptance. The A-C dimension analyzes satisfaction regarding the level of control the family system exerts over its members. The I dimension refers to the subject’s attachment to values (moral, religious, economic, etc.) preferred in the family. The questionnaire has satisfactory indices of internal consistency of scales for all versions, and Cronbach’s alpha is 0.92 (for the specific scales: Com α = 0.88, Coh α = 0.87, A-C α = 0.79, and I α = 0.86). It also has positively validated internal consistency and standards set separately for girls and boys.

### 2.4. Statistical Analyses

All statistical analyses were performed using JASP (version 0.13.1.0) [74]. Descriptive statistics (means, standard deviations, and frequency distributions) were calculated for all primary study variables. Scale reliability was evaluated using Cronbach’s alpha coefficients.

In order to verify the research hypotheses, correlation analyses were performed, followed by stepwise regression for the three loneliness sub-scales. Distribution normality was tested using the Shapiro–Wilk test, and Spearman’s coefficient was used to determine the correlation strength [75].

To identify the best model explaining the feeling of loneliness, the results were divided according to the mean values in the sub-scales into high and low levels of loneliness. The dichotomized results were used to perform logistic stepwise multiple regression analyses. Logistic regression models were selected because preliminary analyses indicated marked skewness in the distributions of the loneliness subscales, violating the assumptions of normality required for linear regression. Dichotomizing the dependent variable (at the sample mean) facilitated the modeling of high- vs. low-risk groups and the direct interpretation of odds ratios. A stepwise regression procedure was implemented due to the high intercorrelations among candidate predictors. This method enabled the selection of the most informative variables, minimizing redundant effects and highlighting those constructs that contribute most substantially to the variance in loneliness outcomes [76].

Due to the marked imbalance in gender distribution, no gender-stratified analyses were conducted. Statistical significance was set at *p* < 0.05 for all tests.

## 3. Results

The results are presented in three main parts: descriptive statistics for all study variables, correlation analyses between the main constructs, and stepwise logistic regression models predicting levels of loneliness. Descriptive statistics (means and standard deviations) and the results of the Shapiro–Wilk normality test for the sample and study variables are shown in Table 1.

In order to identify the direct relations between anxiety (A), specific elements of family relations, such as Com, Coh, A-C, and I, and the feeling of loneliness in adolescence, correlation analysis (see Table 2) was performed.

All correlations were statistically significant at the 0.001 level (Table 2). Strong positive correlations were observed between the loneliness sub-scales: IO and SO (rho = 0.724), IO and BA (rho = 0.686), and SO and BA (rho = 0.725). Loneliness (IO, SO, BA) also showed robust positive associations with anxiety-trait (rho values ranged from 0.460 [BA–A] to 0.623 [IO–A]). Moderate negative correlations were found between all loneliness sub-scales and family relation indices: for example, IO and Com (rho = −0.399), SO and Coh (rho = −0.362), and BA and Identity (rho = −0.366). Anxiety-trait was negatively correlated with all family relations dimensions (A and Com: rho = −0.400; A and Coh: −0.344; A and A-C: −0.467; A and I: −0.383). Strong positive interrelations were also noted among the family relations indices themselves (e.g., Com and Coh: rho = 0.867; Com and I: 0.774).

Logistic regression analyses were performed for each loneliness component to confirm the role of intrafamilial relation indices and anxiety in explaining the feeling of loneliness among adolescents (see Table 3, Table 4 and Table 5).

The best-fitted model explaining the level of the feeling of exclusion (IO) was reported for the anxiety-trait and identity variables (Table 3). The final model, containing both variables, yielded Cox and Snell R^2^ values of 0.249 (only anxiety) to 0.262. Although the change in model fit (ΔΧ^2^ = 2.42, *p* = 0.12) did not reach statistical significance, the inclusion of identity improved the overall explanatory power of the model. Anxiety-trait was a significant predictor (OR = 1.13, 95% CI: 0.07–0.17, *p* < 0.001), while identity did not reach statistical significance (OR = 0.94, 95% CI: −0.14–0.02, *p* = 0.126).

The logistic regression model predicting lack of closeness (Social Others, SO) included anxiety-trait and cohesion as significant predictors (Table 4). The model showed good fit, with a Cox and Snell R^2^ value of 0.267 and significant improvement compared to the simpler model (ΔΧ^2^ = 9.13, *p* = 0.003). Higher anxiety-trait was associated with increased odds of lack of closeness (OR = 1.11, 95% CI: 0.054–0.16, *p* < 0.001), while higher cohesion was associated with lower odds (OR = 0.91, 95% CI: −0.16–−0.03, *p* = 0.004).

The regression analysis was then conducted for the model where the dependent variable was the affiliation level and the predictors were the anxiety-trait and family relationship dimensions (Table 5). The model showed significant improvement over simpler models (ΔΧ^2^ = 9.91, *p* = 0.002) and explained 23.6% of the variance based on Cox and Snell R^2^ values. Higher anxiety-trait was associated with increased odds of high affiliation (OR = 1.11, 95% CI: 0.05–0.15, *p* < 0.001), while lower communication corresponded to decreased odds (OR = 0.84, 95% CI: −0.27–−0.07, *p* = 0.001). Higher autonomy-control was linked to increased odds of high affiliation (OR = 1.17, 95% CI: 0.05–0.27, *p* = 0.003).

Anxiety-trait and the evaluation of family functioning are very strong predictors of loneliness among young people. The models presented have pseudo r-square values between 0.24 and 0.27 using the Cox and Snell statistic, which indicates that there are other predictors not included in our study that may be worth considering in order to improve the match. In each model, anxiety plays the main explanatory role. The role of the specific dimensions of intrafamilial relations in explaining different components of adolescent loneliness is also clearly observable.

## 4. Discussion

The goal of the study was the analysis of the feeling of loneliness among young people and the relationship between loneliness and anxiety as a trait, and the evaluation of elements of intrafamilial relations.

The results of the correlation analyses confirmed that specific aspects of intrafamilial relations—communication, cohesion, autonomy-control, and identity—were significantly associated with adolescent loneliness in each of its dimensions. Higher ratings of communication, cohesion, and identity within family relationships correlated with lower perceived loneliness, whereas lower ratings on these aspects were linked to increased loneliness scores. Anxiety-trait also demonstrated a robust positive association with all facets of loneliness, indicating that adolescents with higher levels of anxiety reported stronger feelings of exclusion, lack of closeness, and reduced affiliation. These findings support hypotheses H2 and H3, highlighting the relevance of both family relational aspects and anxiety as key correlates of adolescent loneliness.

Regression analyses utilizing logistic models were purposefully selected for their ability to identify factors that increase the likelihood of experiencing high loneliness, as defined by a clinically and theoretically relevant threshold. This dichotomization approach was grounded in the need to distinguish adolescents at elevated risk—those whose loneliness scores exceed the population mean—from their less affected peers, thus facilitating clearer interpretation and direct application in prevention and intervention efforts. Logistic regression is particularly suited to this aim, as it allows for the estimation of odds ratios and the identification of distinct risk and protective factors, which is highly valued in both theoretical and applied psychological research. Anxiety-trait emerged as the main predictor across all models, consistently distinguishing adolescents reporting greater loneliness. In addition, individual family relational factors—such as identity, cohesion, and communication—contributed uniquely to the explanation of exclusion, lack of closeness, and affiliation, respectively. The combined models accounted for a meaningful proportion of the variance in loneliness, underscoring that both anxiety and family relationship dynamics play significant roles in explaining differences in loneliness among adolescents. This directly supports hypothesis H1.

The study presented herein has its limitations due to a relatively small sample size and its selection (chosen high schools), which hinders the generalization of the findings. The gender imbalance present in the group also prevented cross-gender analyses. The use of self-report scales may be associated with an increased tendency of participants to provide socially desirable responses. Another questionable aspect is the scale measuring the feeling of loneliness, which, despite good psychometric properties, appears to insufficiently capture the mean level of loneliness. Family relationships were assessed exclusively by adolescents, which may limit the understanding of systemic dynamics. While logistic regression with dichotomized outcomes enabled clear identification of high-risk groups in our study, we recognize that alternative modeling strategies—such as linear regression or generalized linear models—could also reveal nuanced relationships within the data and offer complementary insights into the complexity of adolescent loneliness. It could be beneficial to conduct a qualitative study on loneliness among teenagers, which could provide a better insight into the nature of this phenomenon during this life stage.

The results obtained are consistent with previous studies [30,37,41,42,77,78], verifying the hypotheses posed, and show that anxiety understood as a trait and the way young people rate their family relationships in terms of communication, cohesion, autonomy-control, and identity are strongly connected to feelings of loneliness and all of its aspects. Anxiety-trait is a particularly significant predictor of loneliness. This suggests that adolescents who are prone to anxiety may withdraw, avoid social interactions, and interpret social cues negatively, which in turn can lead to exclusion. The existing data has revealed that elevated levels of anxiety and social anxiety are tied to the feeling of loneliness [42,77,79,80].

The analyses of the specific aspects of loneliness reveal more subtle relations, not only with anxiety but also with the evaluation of intrafamilial relations. The anxiety and identity aspects of family relationships are particularly strongly correlated with the lack of close, intimate relationships with others. This means that teenagers who show higher anxiety are less attached to the values preferred in their families. The values connected to morality, religious, or material systems adopted by their loved ones are not important to them, and young people distance themselves from these values. Their value systems are not based on their family’s value systems. During adolescence, there are many changes occurring in the social functioning of individuals and their expectations of others [28], which may result in undermining family patterns. However, as demonstrated by the results of this study, a lack of foundation within the family value system, together with increased anxiety, is connected to the feeling of loneliness, especially regarding intimate others. It can be assumed that this leads to undermined confidence in relationships, not only with loved ones but also, more generally, with other people, and peers in particular. A young person may have difficulties creating satisfactory interpersonal relations. The results show the subtle and complex nature of this phenomenon. They may confirm that disruptions to the internal relationship pattern passed on by parents contribute to the feeling of loneliness [79]. Negative evaluations of intrafamilial relations in the area of identity may be connected to the lack of acceptance from loved ones [63,64], which additionally increases anxiety. It can be assumed that without a secure family base for integrating and exploring identity, adolescents may lack a point of return, which can increase confusion and emotional distance, not only from loved ones but also from the broader social world.

Another aspect of loneliness related to the lack of closeness is best explained by the anxiety-trait and low scores regarding cohesion in the family. This means that young people who feel anxious and assess their family relationships as cold, stiff, preventing them from sharing feelings, or unsupportive, more often feel that they lack social closeness. Cohesion serves as an indicator of emotional bonding, and low levels of family cohesion are directly linked to a lack of closeness. Adolescents may internalize the notion that distant relationships are risky and generalize this pattern to relationships outside the family. It is known from other research that the levels of parental support and emotional expression are significant determinants of loneliness in adolescence [37]. This suggests that the atmosphere of trust and emotional closeness created by parents is the factor that minimizes loneliness. Some teenagers struggle with loneliness, and they are those who, at the same time, feel more anxious about their environment and evaluate their family relations as negative.

The feeling of loneliness includes not only exclusion and lack of closeness but also poor affiliation, which means that the very sense of loneliness involves the conviction that one cannot build relationships with others and create satisfactory bonds. Other studies confirm that the level of affiliation and social competencies are important aspects of the growing sense of isolation [47]. Anxiety and low-rated communication in the family, together with highly scored control, are strongly correlated with loneliness among young people. Those who are prone to anxiety and are convinced that there is no open communication in their families, but rather stiffness and control dominate, are more vulnerable to loneliness in terms of a lack of belonging and social affiliation. This may result from limited opportunities for social skill development, which are likely hindered by low family communication and high parental control. Without practicing these skills, adolescents may struggle to establish social affiliation and form satisfying relationships. This points to the important role of the family as the environment in which social skills are shaped to support personal development and protect young people from dysfunctional patterns of thinking, feeling, and behaviors. Wide-scale longitudinal research conducted from childhood to early adulthood emphasizes the role of loneliness in psychological well-being of young adults and stresses the role of early intervention [81]. It is known that loneliness facilitates isolation, restricts social interests, and increases shyness, low self-esteem, and uncertainty [46] and, consequently, may lead to a decrease in life satisfaction and an increase in missed life opportunities.

Overall, our findings reveal how anxiety and the quality of intrafamilial relationships jointly contribute to various facets of adolescent loneliness and highlight the subtle, multi-layered nature of these experiences. While loneliness can sometimes serve developmental functions, its intensity and persistence signal a need for early support—particularly for those youth identified as most at risk.

Importantly, the methodological approach adopted in this study not only strengthened the identification of key psychological and family risk factors but also enhanced the practical utility of our results. By distinguishing adolescents at elevated risk for severe loneliness, this technique aligns with real-world needs in education, mental health, and public health policy. Targeted interventions, such as family support programs, social skills training, and individualized counseling, can therefore be more accurately directed to those young people who will benefit most. Furthermore, defining clear risk thresholds is consistent with current public health screening practices, supporting the strategic allocation of resources and the development of preventive strategies aimed at reducing the negative impact of loneliness during this critical stage of life.

## 5. Conclusions

Our analyses confirmed that anxiety-trait and key family relationship factors—such as communication, cohesion, autonomy-control, and identity—are strong predictors of adolescent loneliness, supporting all proposed hypotheses. Higher anxiety and less favorable family dynamics were associated with greater feelings of loneliness in its various dimensions. These findings underscore the joint impact of psychological and relational factors on loneliness among young people.

The results obtained and the analyses performed enable conclusions that developing adequate, early implemented support programs may yield positive results. Unfortunately, so far, there have been no effective methods for reducing the feeling of loneliness [82]. This is most likely due to the high differentiation and complexity of this phenomenon. As indicated by our study, for an intervention to be successful, it should be widespread and include the parents. Some researchers emphasize that efforts focusing on changing the negatively understood concept of loneliness should not only facilitate building bigger social networks but also concentrate on subjective, perceived loneliness [51]. Following this thought, it could be said that it is not only about meeting the basic emotional needs within the family and developing adults’ awareness regarding relationships but also about increasing the understanding of young people of what loneliness is, what its components are, and how they can better cope with it. Raising teenagers’ awareness of the psychological mechanisms of loneliness and developing social skills to enable them to create satisfactory relationships with others could produce positive results.

These conclusions should be interpreted in light of several limitations, including the relatively small and non-representative sample, gender imbalance, and reliance on self-report measures. Future research is recommended to address these methodological constraints, particularly through qualitative approaches and more diverse samples, to gain a deeper understanding of adolescent loneliness.

In summary, this study highlights both the complexity and modifiable nature of adolescent loneliness, pointing to the need for comprehensive, multi-level interventions targeting psychological, relational, and educational factors.

## Figures and Tables

**Table 1:** Descriptive statistics for identified variables.

	IO	SO	BA	A	Com	Coh	A-C	I
N	136	136	136	136	136	136	136	136
M	21.169	8.206	10.493	47.368	29.676	29.11	31.471	29.507
SD	6.58	3.041	3.062	9.736	6.407	7.081	5.908	5.503
Shapiro-Wilk	0.97	0.855	0.944	0.981	0.944	0.949	0.942	0.979
*p*-value of Shapiro-Wilk	0.005	<0.001	<0.001	0.054	<0.001	<0.001	<0.001	0.034
Min	10	5	6	27	11	8	13	13
Max	38	16	20	67	40	40	40	40

**Abbreviations:** IO, Intimate Others; SO, Social Others; BA, Belonging and Affiliation; A, Anxiety-Trait; Com, Communication; Coh, Cohesion; A-C, Autonomy-Control; I, Identity.

**Table 2:** Spearman’s rho correlations for loneliness and anxiety-trait and evaluation of family relations in terms of communication, cohesion, autonomy-control, and identity.

Spearman’s Rho	IO	SO	BA	A	Com	Coh	A-C
SO	0.724 ***	-					
BA	0.686 ***	0.725 ***	-				
A	0.623 ***	0.536 ***	0.460 ***	-			
Com	−0.399 ***	−0.374 ***	−0.374 ***	−0.400 ***	-		
Coh	−0.351 ***	−0.362 ***	−0.335 ***	−0.344 ***	0.867 ***	-	
A-C	−0.318 ***	−0.383 ***	−0.266 **	−0.467 ***	0.717 ***	0.624 ***	-
I	−0.402 ***	−0.420 ***	−0.366 ***	−0.383***	0.774 ***	0.754 ***	0.590 ***

**Notes:** ** *p* < 0.01, *** *p* < 0.001. **Abbreviations:** IO, Intimate Others; SO, Social Others; BA, Belonging and Affiliation; A, Anxiety-Trait; Com, Communication; Coh, Cohesion; A-C, Autonomy-Control; I, Identity.

**Table 3:** Stepwise logistic regression best model predicting Intimate Others (IO) from anxiety-trait and family relations. Higher anxiety significantly increased the odds of reporting exclusion, while identity was not a significant predictor.

Cox & Snell R^2^	ΔΧ^2^	*p*	Parameter	Estimate	SE	z	Odds Ratio	Wald Statistic	df	*p*	95%CI Lower	95%CI Upper
0.262	2.421	0.12	Int.	−4.105	1.951	−2.103	0.016	4.425	1	0.035	−7.930	−0.280
			A	0.12	0.026	4.633	1.128	21.463	1	<0.001	0.069	0.171
			I	−0.063	0.041	−1.528	0.939	2.336	1	0.126	−0.144	0.018

**Notes:** IO level ‘high’ coded as class 1. **Abbreviations:** IO, Intimate Others; A, Anxiety-Trait; I, Identity.

**Table 4:** Stepwise logistic regression best model predicting Social Others (SO) from anxiety-trait and family relations. Both higher anxiety and lower cohesion were significant predictors of SO.

Cox & Snell R^2^	ΔΧ^2^	*p*	Parameter	Estimate	SE	z	Odds Ratio	Wald Statistic	df	*p*	95%CI Lower	95%CI Upper
0.267	9.133	0.003	Int.	−2.94	1.71	−1.719	0.053	2.954	1	0.086	−6.292	0.412
			A	0.105	0.026	4.05	1.110	16.406	1	<0.001	0.054	0.155
			Coh	−0.097	0.034	−2.871	0.908	8.243	1	0.004	−0.163	−0.031

**Notes:** SO level ‘high’ coded as class 1. **Abbreviations:** SO, Social Others; A, Anxiety-Trait; Coh, Cohesion.

**Table 5:** Stepwise logistic regression best model predicting Belonging and Affiliation (BA) from anxiety-trait and family relations. Higher anxiety and lower communication, along with higher autonomy-control, significantly increased the odds of BA.

Cox & Snell R^2^	ΔΧ^2^	*p*	Parameter	Estimate	SE	z	Odds Ratio	Wald Statistic	df	*p*	95%CI Lower	95%CI Upper
0.236	9.909	0.002	Int.	−5.049	2.029	−2.488	0.006	6.192	1	0.013	−9.026	−1.072
			A	0.1	0.025	4.01	1.106	16.078	1	<0.001	0.051	0.149
			Com	−0.17	0.052	−3.263	0.844	10.645	1	0.001	−0.272	−0.068
			A-C	0.161	0.054	2.949	1.174	8.699	1	0.003	0.054	0.267

**Notes:** BA level ‘high’ coded as class 1. **Abbreviations:** BA, Belonging and Affiliation; A, Anxiety-Trait; A-C, Autonomy-Control.

## Data Availability

The data presented in this study are available on request from the corresponding author due to the sensitive nature of the data and ethical considerations related to participant protection.
